# Pathway to developing paediatric non-communicable disease guidelines in Malawi: a scoping review

**DOI:** 10.1186/s12913-025-13410-4

**Published:** 2025-09-03

**Authors:** Dominic Moyo, Megumi Nagase, Amos Msekandiana, Frank Phoya, Takondwa Chimowa, Elizabeth M. Molyneux, Ralf Weigel, Emmie Mbale, Jonathan Chiwanda Banda, Carsten Krüger, Yamikani Chimalizeni

**Affiliations:** 1 Kamuzu University of Health Sciences, Blantyre, Malawi; 2 Witten/Herdecke University, Witten, Germany; 3 Queen Elizabeth Central Hospital, Blantyre, Malawi; 4 Department of Non-Communicable Diseases, Ministry of Health, Lilongwe, Malawi

**Keywords:** Paediatric non-communicable diseases, Guidelines review, Quality of care, Malawi

## Abstract

**Background:**

Non-communicable diseases (NCDs) in children are becoming the leading cause of morbidity and mortality globally. Low- and middle-income countries have not been spared. To improve the quality of care, there is need to use evidence-based and locally relevant interventions. Clinical practice guidelines are essential to provide recommendations that optimise and standardise care. This scoping review was performed to assess the scope, content, and structure of existing paediatric clinical practice guidelines in Africa that can inform the development of paediatric guidelines for NCDs in Malawi.

**Methods:**

Pubmed, Scopus, and Cochrane databases were searched to identify paediatric NCDs guidelines from Africa published between 2003 and 2022 in English. In addition, a manual search of grey literature was conducted online and experts from centres around Africa were contacted. Four independent reviewers analysed the guidelines by answering five specific questions on their scope, structure, content, recommendations and implementation.

**Results:**

A total of 19 guidelines from 13 African countries were included, seven from East Africa, four from Southern Africa and two from West Africa. There were six guidelines specifically for children while the rest were integrated guidelines for both adults and children. There were no specific guidelines for NCDs. However, common NCDs that were covered included non-accidental injuries, developmental disorders, mental health, respiratory, cardiovascular, endocrine, renal, rheumatological and haemato-oncological disorders. There was heterogeneity in how these topics were covered in that the specific paediatric guidelines had more detailed content for children than the integrated guidelines. Most guidelines lacked aspects of prevention and health promotion.

**Conclusion:**

This review evaluated paediatric guidelines from Malawi and the African region to support the development of national paediatric NCD guidelines. The findings highlighted significant gaps in addressing comprehensive aspects, particularly in health promotion and prevention measures, across the 19 reviewed guidelines. Moving forward, the development of paediatric NCD guidelines for Malawi will incorporate insights from African colleagues and address identified content gaps. Addressing these gaps presents an opportunity to tackle the burden of paediatric NCDs.

## Background

The increasing global burden of non-communicable diseases (NCDs) over the past two decades is a well-established trend, resulting in approximately 41 million deaths in adults and children annually, worldwide [1, 2]. Most of these deaths occur in low- and middle-income countries (LMICs) and sub-Saharan Africa is facing a growing NCDs epidemic [2–4]. In 2019, NCDs and injuries accounted for half of the global mortality among children aged 5 to 14 years [4, 5], and this number continues to rise. Despite clear evidence of this emerging pattern, the long-term impact of NCDs on children is often overlooked in global monitoring, goals and priorities [6]. The life-course approach suggests that childhood and early adolescence are a ´global window´ of opportunity to effectively reduce disease risk and to implement cost-effective interventions both within households and the healthcare systems [7–9].

Malawi has a young population with more than half being under 18 years of age [10], and the population is predicted to nearly double by 2050 [11]. Despite the recent success in reducing the burden of infectious diseases (e.g. HIV, tuberculosis, and malaria) and reducing under-five mortality rate [12], the country suffers from the mounting threat of NCDs among the young generation. In 1990, NCDs and injuries accounted for 33 per cent of the entire disability-adjusted life years (DALYs) among 5 to 19-year-olds in Malawi, but by 2019, this rate had risen significantly to 46 per cent [5]. Mental health disorders, neoplasms, asthma, epilepsy and neurological disorders are important contributors to the DALYs [5]. Given that NCDs often go undetected and underreported where poverty prevails and diagnostic equipment is scarce [13, 14], those numbers may be the tip of the iceberg.

To address these health needs, the Malawian government has developed the NCDs Action Plan 2017–2022 [15], with emphasis on prevention, cost-effectiveness, evidence-based strategies and the delivery of essential care across different levels of the health system. However, challenges persist in providing basic care for NCDs. Chronic shortage of drugs and diagnostic equipment are widespread in primary, secondary and tertiary care facilities, and the budget allocated for NCDs is disproportionately constrained by infectious diseases [16, 17]. For paediatric NCDs in particular, the severe shortage of paediatricians, with a ratio of 1 paediatrician to 300,000-500,000 children [18], and outdated resources that are primarily tailored for adults contribute to the problem. Meanwhile, an increasing number of children and adolescents are exposed to new health hazards resulting from rapid urbanisation, lifestyle changes (e.g. alcohol consumption, substance abuse, poor diet, lack of exercise), and environmental conditions affected by climate change [19]. This also jeopardises Malawi’s ability to achieve the child health targets of Sustainable Developmental Goal 3.

The Institute of Medicine defines clinical practice guidelines as systematically developed statements designed to optimize patient care through evidence-based recommendations [20]. However, in resource-limited settings, locally developed guidelines may not always be strictly evidence-based, and often reflect expert consensus and contextual factors such as resource availability and healthcare challenges [21–23]. Despite this, guidelines are essential to aid healthcare professionals and patients in making informed decisions about suitable healthcare [24]. For instance, they promote proven effective treatment that consistently benefits patients, while excluding ineffective ones, and offer updated and standardised recommendations [25, 26]. These features not only improve patient health outcomes but also enhance the quality of the healthcare system as a whole [27–29]. Thus, clinical practice guidelines can be defined as structured recommendations for standardizing healthcare, guiding disease prevention, diagnosis, treatment and management, based on evidence, expert opinion, or policy considerations.

The effective implementation and use of guidelines relies on their applicability to the specific environment in which they are used [27]. Therefore, the varied demands on a healthcare system, availability of equipment and medicine, expertise among healthcare workers and population patterns, need to be reflected in the guidelines [30, 31]. At present, although there are guidelines available from international and national bodies, only a handful focus on the paediatric age group in LMICs. Examples include the guidelines published by the Global Initiative for Asthma (GINASTHMA) [32] and International Diabetes Federation (IDF) [33] that distinctly target children in LMICs to optimise disease management and education, including psychological care and guidance to minimise future risk. Meanwhile, the systematic review of existing epilepsy guidelines conducted by the International League Against Epilepsy (ILAE) revealed that 29 per cent of references did not specify age groups, and only 22 per cent focused on children [34]. Only 1.6 per cent of references identified and included in the review were from Africa, with most coming from North America and Europe. This lack of relevance to the local socio-demographic, cultural and economic aspects of Malawi may result in low adherence and usability [27]. Tailored new guidelines for the Malawian context are therefore essential and entail consolidating relevant, available evidence for the needs of the child population.

To fill the current gap in educational resources and skills training among healthcare workers, the Else Kröner Malawi Child Health Centre was launched within the Paediatrics and Child Health Association of Malawi (PACHA) in August 2022, in collaboration with Kamuzu University of Health Sciences (KUHeS, Malawi) and Witten/Herdecke University (UW/H, Germany). The centre will develop and implement evidence-based, locally relevant paediatric NCD guidelines that integrate disease diagnosis, treatment, prevention and promotion. As such, the primary aim of this review was to assess the structure, content and overall approach of existing paediatric guidelines in Malawi and African region as a baseline to inform newly developed child health NCDs guidelines.

## Methods

This review was conducted according to the Preferred Reporting Items for Systematic Reviews and Meta-Analyses Extension for Scoping Reviews (PRISMA-ScR) [35]. The protocol did not require ethical approval as it utilised publicly available clinical practice guidelines. The funding organisation Else Kröner-Fresenius Stiftung (EKFS) had no influence on protocol development, data analysis and writing of the manuscript.

### Data sources and search strategy

Guidelines and academic literature meeting the eligibility criteria were searched in PubMed, Scopus, Cochrane and relevant NCD-specific associations’ websites: for example, GINASTHMA, IDF, World Child Cancer (WCC), International Society of Paediatric Oncology (SIOP), World Heart Federation (WHF), International Society for Social Pediatrics and Child Health (ISSOP), International Child Neurology Association (ICNA), and World Health Organisation (WHO). In addition, hand searches of grey literature were done on Medbox: The Aid Library [36] and Google platforms using terms “country name, children, non-communicable diseases, treatment guidelines”, and selected centres were contacted to provide guidelines in use in those centres. Ministry of Health websites were not screened systematically for relevant guidelines because they were often outdated or did not provide any additional resources. We ensured that at a minimum, the guidelines which were included had relevant stakeholder involvement and systematic methodology in their respective development as per AGREE II item 8 and 12 [37].The medical subject headings and Boolean operators used in the search strategy for online databases and Google platforms were used as in PubMed. Details are outlined in Table 1.

**Table 1:** Search strategy (Search date: 20th June 2022)

Search line	Search term	Results
#1	Child* OR paediatric OR pediatric OR infant OR adolescen* OR kid* OR neonate*	2,775,419
#2	Africa* OR Sub-Sahara*	27,030
#3	Guidelines OR manual OR standard treatment guidelines OR protocol	94,185
#4	NCDs*	27,914
#5	#1 AND #2	13,265
#6	#3 AND #4	3,689
#7	#5 AND #6	65

(“child“[MeSH Terms] OR “child“[All Fields] OR “children“[All Fields] OR “child s“[All Fields] OR “children s“[All Fields] OR “childrens“[All Fields] OR “childs“[All Fields] OR (“child“[MeSH Terms] OR “child“[All Fields] OR “children“[All Fields] OR “child s“[All Fields] OR “children s“[All Fields] OR “childrens“[All Fields] OR “childs“[All Fields]) OR (“child“[MeSH Terms] OR “child“[All Fields] OR “children“[All Fields] OR “child s“[All Fields] OR “children s“[All Fields] OR “childrens“[All Fields] OR “childs“[All Fields]) OR (“child“[MeSH Terms] OR “child“[All Fields] OR “children“[All Fields] OR “child s“[All Fields] OR “children s“[All Fields] OR “childrens“[All Fields] OR “childs“[All Fields]) OR “kid“[All Fields] OR “kids“[All Fields] OR (“infant“[MeSH Terms] OR “infant“[All Fields] OR “infants“[All Fields] OR “infant s“[All Fields]) OR (“adolescences“[All Fields] OR “adolescency“[All Fields] OR “adolescent“[MeSH Terms] OR “adolescent“[All Fields] OR “adolescence“[All Fields] OR “adolescents“[All Fields] OR “adolescent s“[All Fields]) OR (“infant“[MeSH Terms] OR “infant“[All Fields] OR “infants“[All Fields] OR “infant s“[All Fields]) OR (“infant, newborn“[MeSH Terms] OR (“infant“[All Fields] AND “newborn“[All Fields]) OR “newborn infant“[All Fields] OR “neonatal“[All Fields] OR “neonate“[All Fields] OR “neonates“[All Fields] OR “neonatality“[All Fields] OR “neonatals“[All Fields] OR “neonate s“[All Fields])) AND 2003/01/01:2023/12/31[Date - Publication] AND ((“africa“[MeSH Terms] OR “africa“[All Fields] OR “africa s“[All Fields] OR “africas“[All Fields] OR (“african people“[MeSH Terms] OR (“african“[All Fields] AND “people“[All Fields]) OR “african people“[All Fields] OR “africans“[All Fields] OR “black people“[MeSH Terms] OR (“black“[All Fields] AND “people“[All Fields]) OR “black people“[All Fields] OR “african“[All Fields])) AND “Sub-Saharan“[All Fields] AND 2003/01/01:2023/12/31[Date - Publication]) AND 2003/01/01:2023/12/31[Date - Publication] AND ((((“guideline“[Publication Type] OR “guidelines as topic“[MeSH Terms] OR “guidelines“[All Fields] OR (“practice guideline“[Publication Type] OR “practice guidelines as topic“[MeSH Terms] OR “practice guidelines“[All Fields]) OR ((“ambulatory care facilities“[MeSH Terms] OR (“ambulatory“[All Fields] AND “care“[All Fields] AND “facilities“[All Fields]) OR “ambulatory care facilities“[All Fields] OR “clinic“[All Fields] OR “clinic s“[All Fields] OR “clinical“[All Fields] OR “clinically“[All Fields] OR “clinicals“[All Fields] OR “clinics“[All Fields]) AND (“guideline“[Publication Type] OR “guidelines as topic“[MeSH Terms] OR “guidelines“[All Fields])) OR (“practice guideline“[Publication Type] OR “practice guidelines as topic“[MeSH Terms] OR “clinical practice guidelines“[All Fields])) AND (“reference standards“[MeSH Terms] OR (“reference“[All Fields] AND “standards“[All Fields]) OR “reference standards“[All Fields] OR “standardization“[All Fields] OR “standard“[All Fields] OR “standard s“[All Fields] OR “standardisation“[All Fields] OR “standardisations“[All Fields] OR “standardise“[All Fields] OR “standardised“[All Fields] OR “standardises“[All Fields] OR “standardising“[All Fields] OR “standardization s“[All Fields] OR “standardizations“[All Fields] OR “standardize“[All Fields] OR “standardized“[All Fields] OR “standardizes“[All Fields] OR “standardizing“[All Fields] OR “standards“[MeSH Subheading] OR “standards“[All Fields])) OR (“reference standards“[MeSH Terms] OR (“reference“[All Fields] AND “standards“[All Fields]) OR “reference standards“[All Fields] OR “standardization“[All Fields] OR “standard“[All Fields] OR “standard s“[All Fields] OR “standardisation“[All Fields] OR “standardisations“[All Fields] OR “standardise“[All Fields] OR “standardised“[All Fields] OR “standardises“[All Fields] OR “standardising“[All Fields] OR “standardization s“[All Fields] OR “standardizations“[All Fields] OR “standardize“[All Fields] OR “standardized“[All Fields] OR “standardizes“[All Fields] OR “standardizing“[All Fields] OR “standards“[MeSH Subheading] OR “standards“[All Fields])) AND 2003/01/01:2023/12/31[Date - Publication] AND ((“noncommunicable diseases“[MeSH Terms] OR (“noncommunicable“[All Fields] AND “diseases“[All Fields]) OR “noncommunicable diseases“[All Fields] OR (“non“[All Fields] AND “communicable“[All Fields] AND “diseases“[All Fields]) OR “non communicable diseases“[All Fields] OR “NCDs“[All Fields]) AND 2003/01/01:2023/12/31[Date - Publication]))

In addition to the scoping review described above, two authors (MN and CK) assessed the coverage of the 10 paediatric NCDs with the highest burden in sub-Saharan Africa. The data used for this assessment came from the Global Burden of Disease (GBD) study [38], employing DALYs to measure the disease burden. The analysis focused specifically on the target age group of 0 to 14 years, for both females and males.

### Selection criteria

As explained above, guidelines in our study are defined as structured recommendations aimed at improving patient care through evidence-based practices, expert opinions, and policy considerations. The 10 paediatric NCDs selected here are introduced in the following section. The guidelines that were included met the following criteria: (1) published between 2003 and 2022 (2), from the sub-Saharan African region but were also considered other LMICs with similar sociodemographic profiles to Malawi and (3) aligned with the WHO recommendations or guidelines as evidenced by citations of specific WHO guidelines on particular diseases within the documents.

The guidelines were restricted to those published in English. General paediatric guidelines and country-specific, standard treatment guidelines were considered for review if they contained sections on NCDs applicable to children.

### Selected guidelines themes for review

The guidelines were evaluated according to ten specific themes of paediatric non-communicable conditions; (i), child protection, (ii) developmental disorders, (iii) mental health, (iv) neurological disorders, (v) respiratory disorders, (vi) cardiovascular disorders, (vii) endocrine disorders, (viii) rheumatology (ix) renal disorders, and (x) haemato-oncological disorders. Child protection was included in this review as part of NCDs, specifically addressing child abuse, non-accidental injuries, and children’s rights. These issues are crucial to the overall well-being of children, extending beyond the clinical aspects of health. In Malawi, the Child Care, Protection, and Justice Act acknowledges the importance of these matters. However, there are no specific guidelines dedicated to them within the Act, as they are not categorized as communicable diseases or organ disorders.

### Data extraction and analysis

Screening of the literature for this scoping review was done manually. Four paediatric consultants (DM, AM, FP and TC) who deliver clinical care at central hospitals in the country developed specific questions and a data extraction form. The questions to be answered included: (1) what guidelines are available for NCDs in the paediatric population in Africa (2), what is the specific structure of the available guidelines, specifically looking at the setting (whether applicable for inpatient or outpatient use) and at which level of care (whether primary, secondary or tertiary care) (3), whether the guidelines make specific recommendations on disease promotion, prevention, diagnosis and management at various levels of care (4), who are the end users of the guidelines and the guideline implementation strategy (5), which specific NCDs are covered in the guidelines. The four reviewers each reviewed at least two of the following topics: cardiovascular and haemato-oncological diseases, endocrine and respiratory diseases, renal and rheumatological diseases, neurological, developmental, mental health diseases and child protection. Initial findings of the review were presented to the UW/H-EKFS paediatric NCDs guidelines group for refining and selection of conditions to be included in the final extraction and analysis. Disagreements were resolved by consensus among the four consultants or with the involvement of members of the UW/H-EKFS paediatric NCDs guidelines group.

Narrative synthesis of the guidelines was used to summarise the findings according to the predefined categories and questions set out at the beginning of the extraction exercise. Quality assessment of the guidelines and critical appraisal of the evidence supporting the guidelines were considered to be beyond the scope of the review.

## Results

### Search and screening

**Fig. 1:**
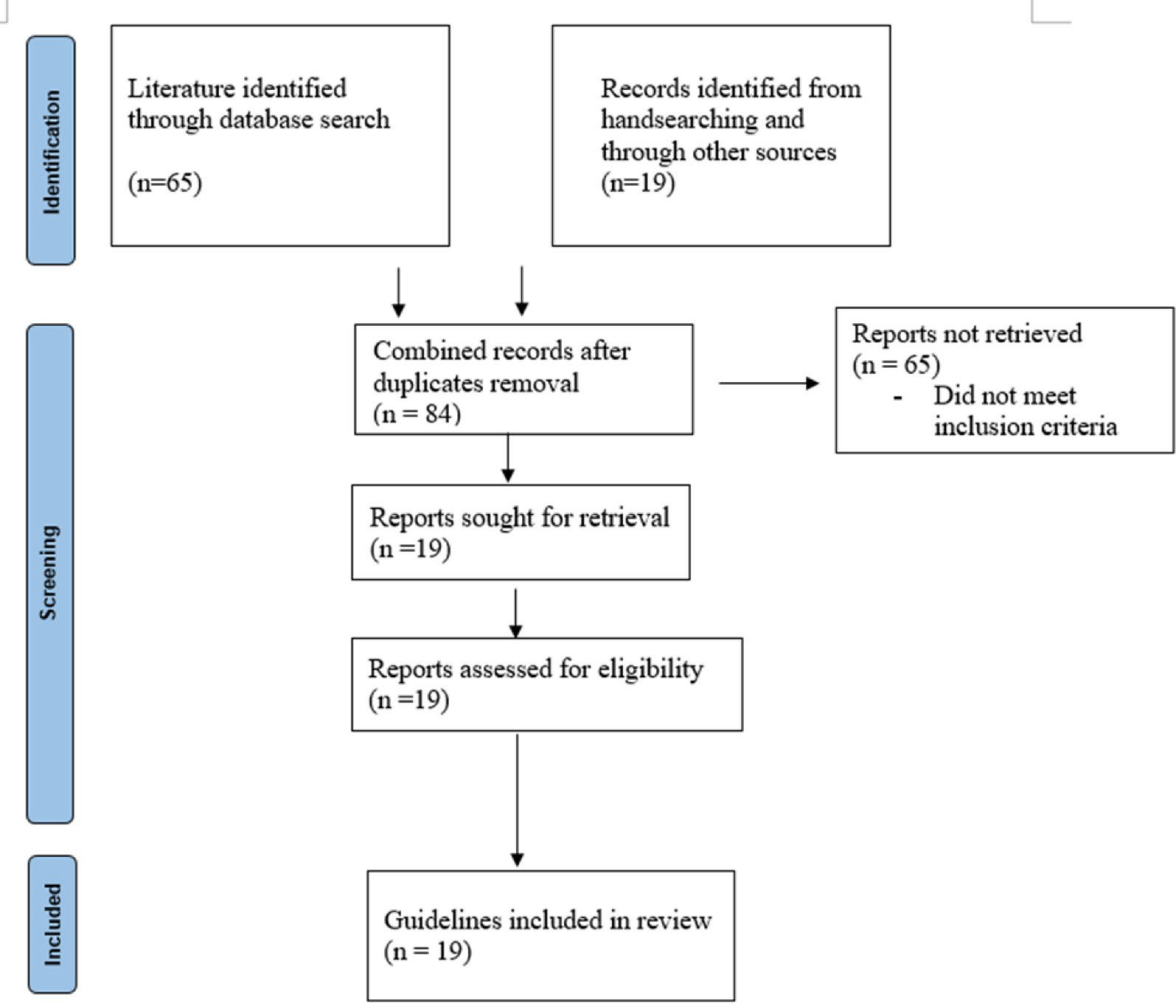
PRISMA flowchart

A total of 84 citations were screened between 7 June 2022 to 29 July 2022 of which 19 were selected for retrieval and full-text review after meeting the eligibility criteria (Fig. 1). The 19 guidelines were from 13 African countries, seven from East Africa, four from Southern Africa and two from West Africa and were published by their respective Ministries of Health. Six guidelines were specifically for children less than 15 years of age, while the rest were integrated guidelines for adults and children.

### Scope and structure of the guidelines

**Fig. 2:**
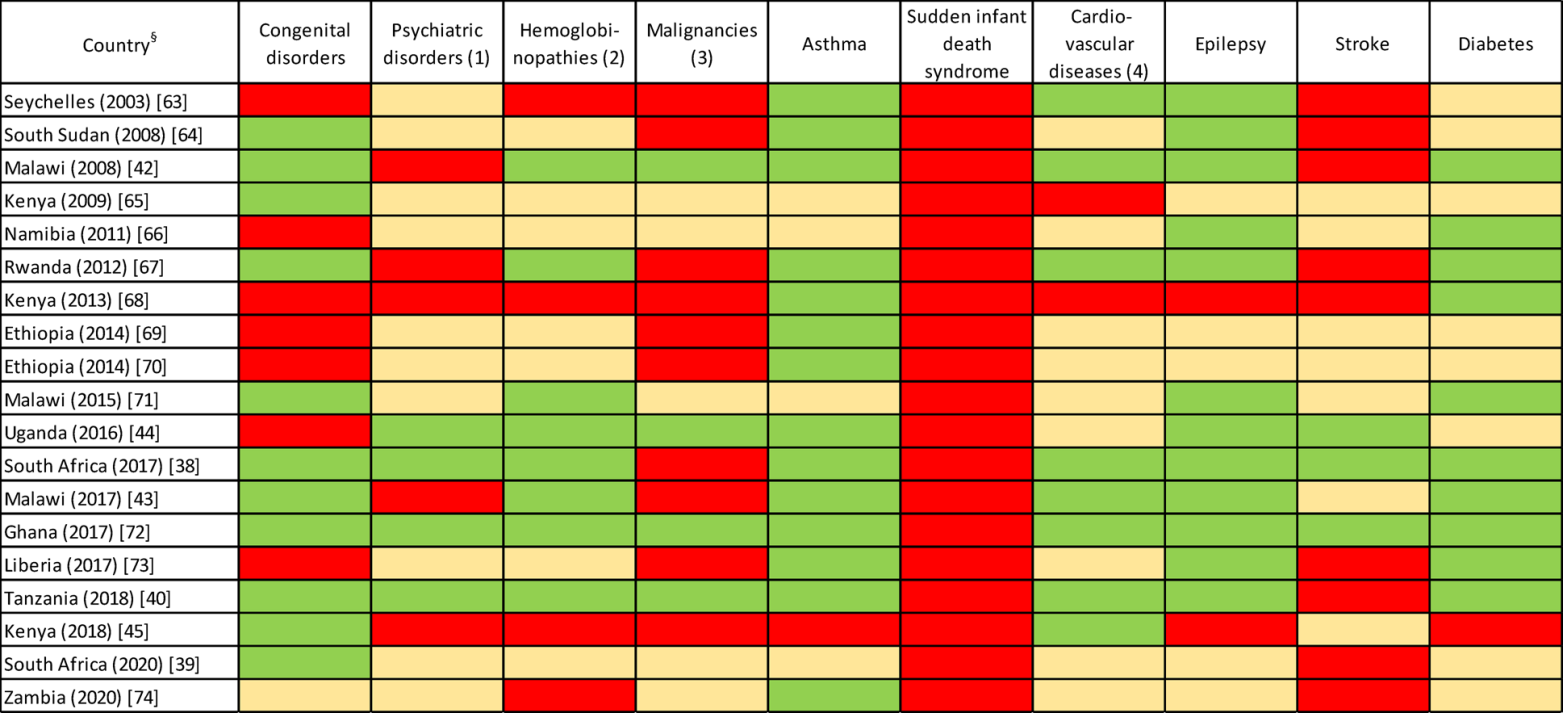
Heatmap - Coverage for the 10 child NCDs with the highest burden of disease (DALYs) in sub-saharan Africa within the reviewed guidelines*# *: Reviewed diseases in the respective category: (1) depression, autism spectrum disorders (2), sickle cell disease, anaemia (3), leukemia, lymphoma, other malignancies (4), rheumatic heart disease, cardiomyopathies #: Assessment grading - green: the disease(s) is (are) covered with focus on children, yellow: the disease(s) is (are) covered but without focus on children or is (are) partly covered, red: the disease(s) is (are) not covered §: figure in brackets – year of publication; figure in square brackets – reference number

Of the 19 reviewed guidelines, their coverage of the 10 child NCDs with the highest burden of disease (DALY) in sub-Saharan Africa is shown in Fig. 2. For age category 0–14 yrs, both sexes, the DALYs for the following 10 major paediatric NCD categories are: congenital disorders 6.38%; psychiatric disorders including depression, autism spectrum disorders, ADHD 1.14%; haemoglobinopathies including sickle cell disease and anaemia 1%; leukemia, lymphoma, other malignancies 0.74%; asthma 0.5%; sudden infant death syndrome 0.43%; cardiovascular disorders including rheumatic heart disease and cardiomyopathies 0.41%; epilepsy 0.41%; stroke (most likely due to sickle cell disease) 0.22%; diabetes mellitus and endocrine disorders 0.2%. Asthma, epilepsy, diabetes and haemoglobinopathies are mostly well covered whereas sudden infant death syndrome is not and congenital disorders and malignancies are insufficiently covered.

The characteristics of the guidelines are summarised in Table 2. Generally, the guidelines provided a brief background, causes or risk factors of the conditions, clinical features (e.g. signs and symptoms and salient examination findings), investigations and both pharmacological and non-pharmacological management. Some guidelines, such as the ones from South Africa [39, 40] and Tanzania [41], included sections on screening and diagnostic criteria for specific conditions, complications, referral criteria and a formulary. Overall, sections on health promotion and prevention of specific disease conditions were poorly covered for all topics except oncology, rheumatology and renal disorders. The integrated guidelines for both adults and children had limited scope and coverage of paediatric NCDs compared to those specifically for children. Several NCDs were not covered in many of the guidelines or covered under other conditions. For example, juvenile idiopathic arthritis was mentioned as an orthopaedic condition, since it is not yet recognised as a major contributor to NCDs in most African countries. With regards to child protection, most guidelines referred to local child law legislation which varies from country to country and focused on sexual abuse but lacked advice for other forms of child abuse.

**Table 2:** Characteristics of the 19 guidelines from 13 African countries

Year	Country/Organisation	Title	Scope	Paediatric NCDs included*	Level of care	Setting	Implementation	Recommendations^#^
2003	Seychelles	Standard Treatment Guidelines [63]	- Adults and children - Systems approach	Yes iii, iv, v, vi, vii, ix	All levels	In- and out- patients	All health care workers	Promotion: little Prevention: little Diagnosis: some Management: some
2006	Southern Sudan	Prevention and Treatment Guidelines for Primary Health Care Centres and Hospitals [64]	- Adults and children - Include formulary (essential medicines list)	Yes iii, iv, v, vi, vii, viii, ix	All levels	In- and out- patients	All health care workers	Promotion: some Prevention: good Diagnosis: some Management: some
2008	Malawi	Paediatric Handbook for Malawi [43]	- Paediatrics - Systems, symptoms and disease specific - Includes formulary, growth charts and procedures	Yes i, ii, iv, v, vi, vii, viii, ix, x	All levels	In-patients	Clinicians	Promotion: some Prevention: good Diagnosis: some Management: good
2009	Kenya	Clinical Guidelines for Management of referral of common conditions at level 1: community [65]	- Adults and children	Yes i, ii, iii, iv, v, vi, vii, viii	Community	Out-patients	Community Health Workers	Promotion: good: Prevention: good Diagnosis: little Management: very little
2011	Namibia	Namibia Standard Treatment Guidelines [66]	- Adults and children - Large section on accidents and trauma	Yes ii, iv, v, vi, vii, viii, ix, x	All levels	In- and out- patients	All health care workers	Promotion: good Prevention: good Diagnosis: some Management: some
2012	Rwanda	Paediatric Clinical Treatment Guidelines [67]	- Paediatrics	Yes iii, iv, v, vi, vii, viii, ix	Secondary and tertiary level	In- and out- patients	All health care workers	Promotion: none Prevention: none Diagnosis: good Management: good
2013	Kenya	Basic Paediatric Protocols for ages up to 5 years [68]	- Paediatrics	Yes iv, v, vi, vii, viii	All levels	In- and out- patients	All health care workers	Promotion: none Prevention: very little Diagnosis: some Management: some
2014	Ethiopia	Standard Treatment Guidelines for Primary Hospital [69]	- Adults and children - Systems approach	Yes iii, iv, v, vi, vii, viii, ix	Primary care	Out-patients	Clinicians	Promotion: none Prevention: little Diagnosis: little Management: good
2014	Ethiopia	Standard Treatment Guidelines for General Hospital [70]	- Adults and children	Yes iii, iv, v, vi, vii, viii, ix	Secondary and tertiary care	In- and out-patients	All health care workers	Promotion: none Prevention: little Diagnosis: some Management: some
2015	Malawi	Malawi Standard Treatment Guidelines [71]	- Adults and children - Includes formulary (essential medicines list)	Yes iii, iv, v, vi, vii, viii, ix, x	All levels	In- and out- patients	All health care workers	Promotion: none Prevention: little Diagnosis: some Management: some
2016	Uganda	Uganda Clinical Guidelines [45]	- Adults and children - Use systems approach + with some specific diseases - Includes drug formulary (essential medicines list)	Yes ii, iv, v, vi, vii, viii, ix, x	All levels	In- and out- patients	Targets different cadres - doctors, nurses, community health workers	Promotion: little Prevention: some Diagnosis: some Management: some
2017	South Africa	Standard Treatment Guidelines and Essential Medicines List for South Africa (Hospital level paediatrics) [39]	- Paediatrics - Uses systems approach and includes emergencies and trauma - Includes formulary (essential medicines list)	Yes (i), ii, iii, iv, v, vi, vii, viii, ix, (x)	All levels	In- and out- patients	All health care workers	Promotion: little Prevention: some Diagnosis: good Management good
2017	Malawi	Protocols for the management of common childhood illnesses in Malawi (Hospital) [44]	- Paediatrics - Uses systems and disease specific approach - Included formulary (essential medicines list)	Yes i, ii, iii, iv, v, vi, vii, viii, ix	All levels	In- and out- patients	All health care workers	Promotion: little Prevention: little Diagnosis: good Management: good
2017	Ghana	Standard Treatment Guidelines [72]	- Adults and children - Uses both systems, symptoms and disease specific approach	Yes i, iii, iv, v, vi, vii, viii, ix, x	All levels	In- and out- patients	All health care workers	Promotion: little Prevention: little Diagnosis: good Management: good
2017	Liberia	National Standard Therapeutic Guidelines and Essential Medicines List [73]	- Adults and children - Uses systems and disease specific approach - Includes formulary	Yes iii, iv, v, vi, vii, viii, ix, (x)	All levels	In- and out- patients	All health care workers	Promotion: none Prevention: little Diagnosis: little Management: moderate
2018	Tanzania	Standard Treatment Guidelines and Essential Medicines List for Children and Adolescents [41]	- Paediatrics - Combination of systems, symptoms and disease specific - includes formulary	Yes ii, iii, iv, v, vi, vii, viii, ix, x	All levels	In- and out-patients	All health care workers	Promotion: some Prevention: some Diagnosis: some Management: moderate
2018	Kenya	Kenya National Guidelines for Cardiovascular diseases management [46]	- Adults and children	Yes vi	All levels	In- and out- patients	All health care workers	Promotion: some Prevention: some Diagnosis: good Management: good
2020	South Africa	Standard Treatment Guidelines and Essential Medicines List for South Africa (Primary Healthcare level) [40]	- Adults and children - Uses systems, symptoms and disease specific approach	Yes i, iii, iv, v, vi, vii, viii, (x)	Primary level	In- and out- patients	All health care workers	Promotion: moderate Prevention: moderate Diagnosis: little Management: some
2020	Zambia	Standard Treatment Guidelines [74]	- Adults and children - Includes formulary (essential medicines list)	Yes iii, iv, v, vi, vii, viii, ix, x	All levels	In- and out- patients	All health care workers	Promotion: some Prevention: good Diagnosis: moderate Management: moderate

*: Categories: (i) child protection, (ii) developmental disorders, (iii) mental health disorders, (iv) neurological disorders, (v) respiratory disorders, (vi) cardiovascular disorders, (vii) endocrine disorders, (viii) renal disorders, (ix) rheumatological disorders, and (x) haemato-oncological disorders

#: Assessment grading – none: not present; very little: few short remarks; little: short remarks on some aspects; some: coverage of some aspects; moderate: more extensive coverage of the aspects; good: sufficient, appropriate coverage

All the guidelines were available as soft copies, but without quick links to chapters or topics it was sometimes a lengthy process to find a topic of immediate interest.

### Guidelines implementation and use

The preface of most guidelines indicated that they were for use at various levels of health care and applicable to both inpatients and outpatients, but the guidelines were mostly skewed towards clinical case management at secondary and tertiary facility level, with suggestions for referral to a higher level of health care for specific trigger clinical signs and symptoms. The Standard Treatment Guidelines and Essential Medicines List for South Africa - Hospital Level Paediatric Guidelines [39] were the most comprehensive guidelines among those reviewed.

### Child protection

In most sub-Saharan African countries, legal frameworks exist in the form of a Children´s Act (named differently in different jurisdictions) that define the necessary measures to be taken for children at risk. The acts/legal instruments are applicable at all levels of the healthcare system and intended for use by all healthcare workers involved in child care to ensure a multidisciplinary approach.

### Developmental, mental and neurological disorders

The guidelines for these disorders were primarily relevant at secondary and tertiary health care level and directed towards clinical management. For neurological disorders such as epilepsy most local guidelines are based on guidelines of the International League Against Epilepsy [42]. The guidelines for Tanzania [41], South Africa [39], and Malawi [43, 44] include sections addressing cerebral palsy. The Malawian Paediatric Handbook [43] and the South African Paediatric Hospital Level Standard Treatment Guidelines [39] address autism spectrum disorders. The Tanzanian [41] and South African guidelines [39, 40] cover attention deficit hyperactive disorder (ADHD). Guidelines addressing anxiety, depression, and post-traumatic stress disorder (PTSD) were available in South Africa [39, 40], Uganda [45], and Tanzania [41]. On the other hand, none of the reviewed guidelines within Africa cover global developmental delay and intellectual disability. Likewise, aspects of promotion and prevention were hardly addressed.

### Cardiovascular disorders

Guidelines variously include critical examination findings, diagnostic criteria and a section on when to refer. Kenya has separate comprehensive guidelines for cardiovascular diseases [46] which include common paediatric problems and a section on newborn screening for congenital cardiac diseases. The South African guidelines [39] address cardiac emergencies and common arrhythmias. Most guidelines use ICD-10 coding for each disease. They generally employ umbrella categories rather than specific diagnoses, except for the more common conditions such as acute rheumatic fever, rheumatic heart disease, and tetralogy of Fallot. These guidelines primarily focus on inpatient clinical management. Kenya, notably, has a separate guideline for use in the community that outlines when and how to refer children with specific conditions and includes community education, promotion and prevention.

### Rheumatological and renal disorders

There is a predominant focus on arthritis, with limited coverage of other rheumatic conditions. The guidelines are for a clinical setting, both in and out-patient and due to the specialised nature of various tests required, most guidelines are applicable to the tertiary setting. Recommendations encompass both pharmacological and non-pharmacological interventions for diagnosis and management, providing comprehensive guidance for healthcare professionals in these areas, specifically tailored for implementation in tertiary care clinical settings.

### Respiratory diseases

Asthma was found to be the primary NCD discussed in all the African guidelines that concentrated on diagnosis and effective case management. Preventive measures are typically integrated into the long-term management strategy outline in the guidelines.

### Haematology and oncology

The guidelines are for clinical use in hospitalised patients. Although applicable at the tertiary level, most guidelines do not offer specific details on chemotherapy protocols. Oncology guidelines are often adapted from the national comprehensive cancer network. Sickle cell disease guidelines are developed by a team of experienced clinicians and researchers with input from local authorities. While cancer awareness resources are available, they are not explicitly included as part of the guidelines. Most guidelines provide a brief overview of prevention strategies.

### Endocrine disorders

The guidelines focus on tailored clinical case management and provide a series of prompts to assist clinicians. The main objectives are to prevent complications associated with underlying conditions, such as providing dietary recommendations for Type 1 diabetes. Overall, these guidelines are designed to aid clinicians in managing patients at the secondary or tertiary level of care, both in hospital and outpatient settings.

## Discussion

The review included 19 guidelines from 13 African countries to inform the development of Malawian guidelines for NCDs in children. The selected guidelines generally had sections on background, causes, clinical features, investigations, and management, but often lacked comprehensive coverage of health promotion and preventive measures. Most guidelines were available digitally but lacked quick links to specific topics. They were skewed towards clinical case management at secondary and tertiary levels of healthcare, potentially neglecting primary care needs.

During the search of guidelines, we became aware that there is either remarkable inaccessibility or unavailability of guidelines specifically tailored to the paediatric population and also to the demands of the region/country [27]. Firstly, there is a scarcity of paediatric guidelines that adequately cover NCDs on the continent, in contrast to the greater availability of guidelines that primarily address infectious diseases such as HIV/AIDS, tuberculosis and malaria. Secondly, while the majority of health facilities have guidelines tailored to their specific centers, they are often not readily accessible beyond their respective centers. This limits opportunities for individuals seeking relevant sources to become aware of the existence of such guidelines and to access potentially valuable information. Furthermore, restricting knowledge sharing among regions or countries could negatively impact users of guidelines. They might find differences in knowledge and practices depending on where they are. Additionally, organisations or instituations may struggle to utilise existing literature when creating new guidelines [27]. Lastly among the 19 guidelines reviewed, seven were published more than 10 years ago (before 2014). This may mean that some of them do not reflect current practices and may be outdated [47]. As a result, healthcare workers may be guided to use treatments that do not produce the optimal outcomes [26].

Guidelines are crucial for facilitating effective healthcare by providing a standardised approach to care delivery tailored to specific country or regional resources [25]. They also serve as valuable tools for improved and cost-effective healthcare [48–52]. However, as noted in the results section, the guidelines’ predominant focus on adults, particularly in the established, national treatment guidelines, restricts their usefulness for child health professionals. Even when guidelines cover both adults and children, they may lack sufficient detail on paediatric diseases, limiting the guidelines´ benefits for the paediatric population. Further, particularly for paediatric NCDs services, such limitations could influence resource allocation, which has a high potential to improve quality of care.

The reviewed guidelines also overlook some aspects of the care delivery pathway, as well as components of health promotion and prevention. A care delivery pathway includes seamless care from community level to tertiary or quaternary levels. Illustrating this pathway in guidelines gives healthcare workers a clear coordinated plan to follow and helps providers at different facility levels by defining their roles [53, 54]. Consequently, patient care is consistent and transfer of patients between different levels of care facilities is smooth [53, 55, 56]. Moreover, integration of health promotion and prevention strategies into guidelines can foster a more holistic approach to patient care, that focuses not only on treating current conditions, but also on preventing future illness and complications and comorbidity [57–59]. For instance, guidelines may outline how to prevent childhood obesity as a significant risk factor for future conditions like diabetes mellitus and hypertension and offer evidence-based health promotion interventions. Providing guidance on these matters, including facilitating early screening and detection to patients can serve as a proactive measure to mitigate and prevent future health risks.

### Recommendations

Based on the review, future paediatric NCDs guidelines should consider to:

- Outline a service delivery model for every condition at each level of care, specifying the activities to be carried out at that level.
- Add the transition of care for children into adult-oriented care and determine the appropriate age/state.
- Focus on care delivery measures, as well as include a broad range of allied health services and adopt a dynamic view of the healthcare system through delineation of the roles of various allied healthcareworkers and services.
- Emphasise quality improvement and develop accompanying indicators (to ensure that routine monitoring of disease trends and outcomes is carried out).
- Include comprehensive topic-specific public health measures, such as prevention, screening, and health promotion for every topic.

### Strengths and limitations

To our understanding, this is the first scoping review on paediatric guidelines in sub-Saharan Africa, and will inform the development of country-specific paediatric NCDs guidelines in Malawi. The review and the recommendations for new paediatric NCDs guidelines address the demand for guidelines that are tailored to a specific local environment in sub-Saharan Africa, but are potentially useful to a wider audience given the scarcity of locally developed guidelines in most LMICs [27, 60, 61]. Close alignment with the local contexts may improve the quality of care and health outcomes for patients [62]. In addition, the review conducted by local paediatric experts, safeguards a thorough understanding of the subject and identifies areas for improvement in guideline development.

However, the review has limitations. Guidelines written in English exclude guidelines published in other languages, such as French, which is in common use in West Africa. Although hand searches of grey literature were carried out, relying solely on Google platforms and contacting selected centres, but not including scientifically approved search tools for screening, may not capture all relevant grey literature. Lastly, including only guidelines published in the last 20 years may result in missing older guidelines that may still contain valuable information.

## Conclusion

We reviewed 10 topics of paediatric NCD conditions mainly from grey literature that covered a wide range of NCD topics. The majority of guidelines were hospital and/or clinic-oriented and mainly applicable to secondary or tertiary level facilities. Many guidelines combined the care of adults and children with only a third (31%) focusing solely on paediatrics. Nearly all the guidelines failed to include public health aspects and had little or no information on health education, promotion and prevention. By incorporating these comprehensive aspects across the entire spectrum of childhood NCDs, guidelines could have a significant impact on reducing the burden of these conditions. Nevertheless, most of the guidelines took a multidisciplinary approach to managing various clinical conditions and offered referral options to the next level of care where applicable. Thus, the development of paediatric NCD guidelines for Malawi will benefit from the experience and output of our African colleagues and any gaps in content will be carefully considered.

## Data Availability

No datasets were generated or analysed during the current study.

## Abbreviations

ADHD: Attention deficit hyperactive disorder
AGREE II: Appraisal of Guidelines for Reseach and Evaluation II
DALYs: Disability-Adjusted Life Years
EKFS: Else Kröner-Fresenius-Stiftung
GBD: Global Burden of Disease
GINASTHMA: Global Initiative for Asthma
ICNA: International Child Neurology Association
IDF: International Diabetes Federation
ILAE: International League against Epilepsy
ISSOP: International Society for Social Paediatrics and Child Health
LMICs: Low-and-Middle-Income Countries
NCDs: Non-Communicable Diseases
PACHA: Paediatric and Child Health Association of Malawi
PRISMA-ScR: Preferred Reporting Items for Systematic Reviews and Meta-Analyses
PTSD: Post-traumatic stress disorder
SIOP: International Society of Paediatric Oncology
UWH: Witten/Herdecke University
WCC: World Child Cancer
WHF: World Heart Federation
WHO: World Health Organization
